# *In Vitro* Maturation of Germinal Vesicle Oocytes in Stimulated Intracytoplasmic Sperm Injection Cycles

**Published:** 2011-08-24

**Authors:** Mir Mehrdad Farsi, Seyed Gholam Ali Jorsaraei, Sedigheh Esmaelzadeh, Mohammad Jafar Golaipour

**Affiliations:** 1. Fatemeh Zahra Infertility and Health Reproductive Research Center, Babol University of Medical Sciences, Babol, Iran; 2. Department of Embryology, Gorgan University of Medical Sciences, Gorgan, Iran

**Keywords:** Immature Oocyte, IVM, ICSI

## Abstract

**Objective:**

This study evaluated* in vitro* maturation (IVM) of oocytes in the germinal vesicle (GV) stage in stimulated intracytoplasmic sperm injection (ICSI) cycles.

**Materials and Methods:**

A total of 26 women, aged 18 -37 years, who were candidates for ICSI at the Fatemeh Zahra Infertility and Health Reproductive Research Center in 2007 were recruited for this study. We used the standard long protocol for ovarian stimulation. Follicles >11 mm were punctured 36-38 hours after administration of 10000 IU human chorionic gonadotrophin (hCG). Immature oocytes were cultured for 24-30 hours. Oocytes that liberated polar bodies were injected by sperm prepared within the previous day. IVM fertilized oocytes were cultured an additional 24-30 hours for cleavage. The rates of maturation, fertilization and cleavage in IVM oocytes were recorded and statistically compared to *in vivo* matured sibling oocytes.

**Results:**

There were 279 collected oocytes (mean±SD: 10.73 ± 6.2), of which 4.08±2.79 were subjected to IVM. An average of 2.73 ± 2.15 GV oocytes (70%) developed to metaphase II (MII). Although the maturation rate significantly differed between the IVM and in vivo MII sibling oocyte groups (p=0.027), the numbers of fertilized oocytes (p=0.795) and cleaved embryos (p=0.529) were not significantly high in the *in vivo* group. Transfer of IVM embryos occurred in only three cases with one pregnancy that resulted in the delivery of a healthy baby.

**Conclusion:**

This study shows that culturing GV oocytes can produce acceptable numbers of four-cell embryos on the transfer day. The developmental competence of oocytes is not significantly different between early stage IVM and* in vivo* sibling embryos.

## introduction

Use of external gonadotrophins increases the numbers of harvested oocytes and enhances the pregnancy rate in assisted reproductive technology (ART). Nevertheless, about 15-20% of collected oocytes are immature and, in some cases, germinal vesicle (GV) oocytes may comprise a larger part of retrieved oocytes (1). Extensive studies have been performed on *in vitro* maturation (IVM) of immature oocytes in stimulated or unstimulated cycles (2-4). In unstimulated cycles there is no external gonadotrophin administration, which might be beneficial for avoiding ovarian hyperstimulation syndrome (OHSS). Chian et al. have reported significant improvements in IVM rate by human chorionic gonadotrophin (hCG) priming before the retrieval of immature oocytes from polycystic ovarian syndrome (PCOS) patients (5). According to their study, 78.2% of immature oocytes matured to metaphase II (MII). In order to achieve an adequate number of oocytes, the use of gonadotrophins is routine in stimulated intracytoplasmic sperm injection (ICSI) cycles. Retrieved cumulusoocyte complexes are denuded to access nuclear maturation and possible subsequent injection. Oocytes with GV or those lacking polar bodies undergo further culturing to attain matured MII oocytes. However, the efficacy of *in vitro* matured GV and MI oocytes for clinical use is questionable (1). There are reports of approximately 25% meiotic abnormalities in GV immature oocytes with an IVM rate of 42.9% (6). Despite the possibility that IVM oocytes would not have developmental competence as expected with *in vivo* oocytes, immature oocytes may provide an opportunity for patients with inadequately matured oocytes or those lacking oocytes on the puncture day. Is there a chance for embryo transfer using these immature oocytes? To answer this question we cultured GV oocytes for 24-30 hours and evaluated the rates of maturation, fertilization and cleavage in comparison with *in vivo* matured sibling oocytes from the same cycles.

## Materials and Methods

This clinical study was carried out in the Fatemeh Zahra Infertility and Health Reproductive Research Center from April 2006 until March 2007. The Center’s Ethical Committee approved the study. In total, 26 women aged 18-37 years with at least two collected GV oocytes on the puncture day were included in this study. There were no other excluding factors. Patients were given full information about the study and signed informed consents.

All patients underwent the standard long protocol of pituitary suppression with gonadotrophin releasing hormone (GnRH). After 17 days of oral contraceptives, pre-treatment buserelin subcutaneous injections (Superfact; Hoechst, Germany) were administrated at daily doses of 0.5 cc until the initiation of human menopausal gonadotrophin (HMG; Pergonal; Serono, Italy) on the second or third day of the next cycle. On the day of HMG initiation, the dose of buserelin was modified to 0.25 cc until the time of HCG injection. Patients received HMG at a daily dose of 150-300 IU for 6-7 days, which was modified according to the response. Patients underwent transvaginal ultrasound. When the largest measured follicle(s) reached a maximum mean diameter of 18-19 mm, 10000 IU HCG (Pregnyle, Darupakhsh, Iran) was administrated intramuscularly. Follicles larger than 11 mm were transvaginally punctured 36-38 hours after HCG injection. The oocytes after a maximum of one hour incubation in 6% CO_2_ and 37℃ (Binder, Germany) under mineral oil (Nidoil, Nidacone, Sweden) were denuded with 1% hyaluronidase enzyme (Sigma Aldrich Co., Germany) and a hand drawn glass pipette. With the use of an invert microscope (TE 300, Nikon) at ×200 magnification, GV oocytes were detected. Our criteria for selected GV oocytes were the presence of a prominent nucleus in a homogenous cytoplasm with any type of defect in the oocyte's overall appearance. The selected GV oocytes were placed in a drop of 20-30 µl GΙ media (Vitrolife, Sweden) in separate dishes for 24-30 hours. Oocytes with liberated polar bodies were injected by sperm that were provided during the previous day. Injected IVM oocytes were assessed after 16-18 hours for the appearance of pronuclei based on our routine practice. Zygotes were cultured for an additional 24-30 hours. A maximum of three embryos at the four-cell stage were transferred to each patient. In only three cases, due to unavailable embryos, we used the IVM-produced embryos.

Data that included the rates of maturation, fertilization and cleavage of *in vitro* and *in vivo* matured oocytes were analyzed with SPSS version 18 software. We used the Wilcoxon test to compare groups at the 0.05 level of significance.

## Results

**Fig 1:**
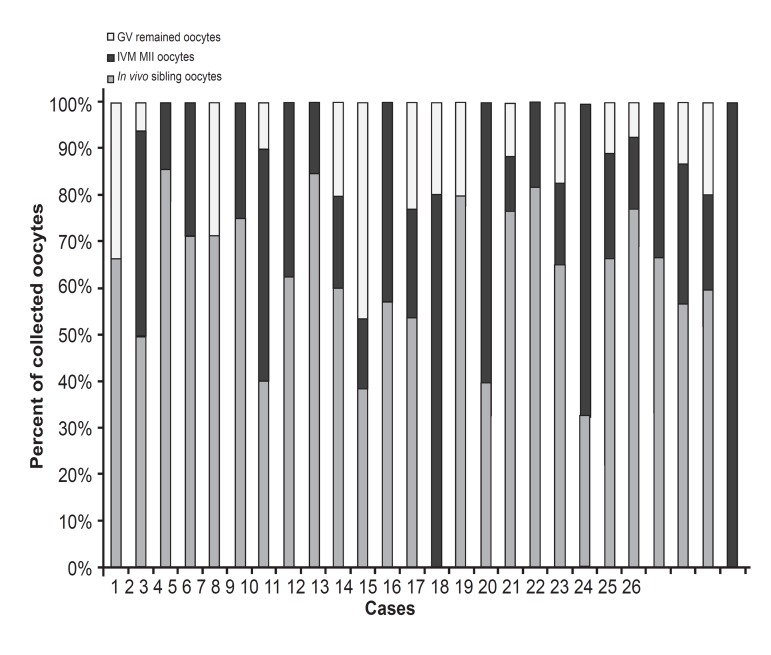
Frequency of oocytes in study cases

A total of 279 oocytes were collected from 26 women who were ICSI candidates. The frequency of harvested oocytes ranged from 2-30. The mean ± SD of oocytes was 10.73 ± 6.2. Of these, 4.08 ± 2.79 (42%) were subjected to IVM (Table 1). In seven cases, GV oocytes included ≥50% of the collected oocytes, with two cases that did not have MII (Fig 1).

**Table 1:** Comparison of collected oocytes, fertilization and cleavage between *in vitro* and *in vivo* sibling matured oocytes


	IVM oocytes	In vivo sibling oocytes	p value
	Mean ± SD (%)	Mean ± SD (%)	
Oocytes	4.08 ± 2.79 (0.42 ± 0.22)		
MII	2.73±2.15 (0.70 ± 0.34)	6.65 ± 4.35 (0.58 ± 0.22)	0.027^*^
Zygotes (2PN)	1.69 ± 1.69 (0.65 ± 0.42)	4.42 ± 3.28 (0.65 ± 0.29)	0.795
Four-cell embryos	1.19 ± 1.36 (0.70 ± 0.38)	2.88 ± 1.9 (0.74 ± 0.29)	0.529


Statistical analysis with Wilcoxon test. *p<0.05 is at the level of significance.

**Fig 2:**
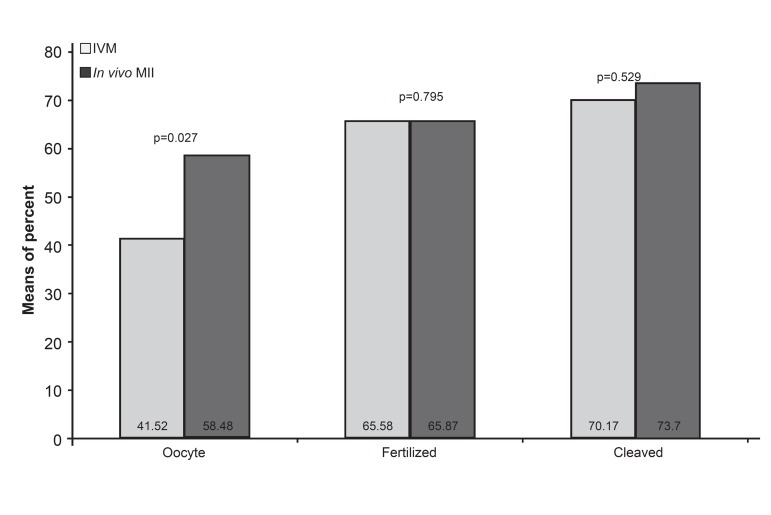
Comparison between means of percent (%) in IVM and in vivo sibling groups

In eleven cases, all retrieved GV oocytes developed to the MII stage after 24-30 hours of incubation. An average of 2.73 ± 2.15 GV oocytes (~70%) developed to MII. The mean ± SD for *in vivo* MII sibling oocytes was 6.65 ± 4.35 (Table1). Comparison between means of percent (%) in IVM and *in vivo* sibling oocyte groups was significantly different (p=0.027, Fig 2). Fertilization occurred in 65% of IVM-MII oocytes with the same percent for *in vivo* MII sibling oocytes (Table 1 , Fig 2). Overall cleaved embryos in the four-cell stage were 70% in the IVM group (average: 1.19). In the *in vivo* sibling group, 74% of embryos were in the four-cell stage (average: 2.88). The differences between means of fertilized oocytes and four-cell embryos in IVM and *in vivo* sibling oocyte groups were not significant (Table 1 , Fig 2).

VM four-cell embryos were transferred in only three patients, with one pregnancy that resulted in the delivery of a healthy baby.

## Discussion

In this study, we evaluated the rates of maturation, fertilization and cleavage in IVM-GV oocytes with *in vivo* matured sibling oocytes collected during the same cycles. It was expected that the culture of GV oocytes for 24-30 hours could provide more embryos for transfer, particularly in cases with few or no mature oocytes available on the puncture day. Successful pregnancies from such prolonged cultures have been reported (7-9).

The percent of GV oocytes in our study (42%) was comparable with a report by Reichman et al. (1) where 37.2% of collected oocytes were in the GV stage. In other studies, the percent of collected GV oocytes was lower than our study, which probably resulted from different study conditions (Table 2). The majority of GV collected from stimulated ovaries liberate the first polar body (PB) spontaneously after a minimum of 22 hours (10). An optimal ICSI time after the first PB extrusion of oocytes has been proposed by Hyun et al. (11). According to their study, GV oocytes would need at least one hour to complete nuclear maturation after the first PB extrusion.

The fertilization rate was 15.8% in oocytes injected within one hour vs. 80% in those injected within one to two hours after the first PB extrusion (11).

**Table 2:** Outcome of IVM cycles from literature


Authors(year)	No. of GV oocytes	*In vitro* maturation rate (%)	Fertilization rate (%)	Cleavage rate (%)	Clinical pregnancy rate/embryo transfer (%)	Live births
Nagy et al. (1996)	14	64	78	71	100	1
Kim et al. (2000)	168	66.7	51.8	84.5		
Yoon et al. (2001)	506	74.3	72.6	89	17.6	9
Shu et al. (2007)		37.5	44.2	100	2	
Reichman et al. (2010)		35.1	60.0	20.5	none	none
Escrich et al. (2010)	131	74.8	59.7			


We cultured GV oocytes for 24-30 hours based on IVM studies (12). Our maturation rate of GV approximated the upper limit of retrieved oocytes reported in stimulated cycles, which was 35%-78% (1, 3, 10, 13). The means of MII oocytes between the IVM and *in vivo* sibling oocytes were significantly different in our study, which was similar to other researches (1, 10).

Fertilization rates in GV-matured and *in vivo* MII sibling oocytes were not significantly different in our study, which confirmed the report of Reichman and colleagues (62.1% vs. 64.0%, p=0.9909). Kim et al. studied two groups of GV oocytes with or without cumulus cells and reported that the presence of cumulus cells did not significantly change the proportion of GV oocytes that reached MII. Overall, the normal fertilization rate was 51.8% (13). In the reports of Escrich et al. (10) and Nagy et al. (7), the rates of fertilization were 59.7% and 78%, respectively. In other studies the fertilization rates in the IVM and *in vivo* groups significantly differed, which was in contrast to our study (10, 13).

PB extrusion and activation of MII oocytes in the subsequent entrance of spermatozoa in fertilization could be defined as oocyte competency. It has been reported that morphological and morphometric measures, and the chromatin condensation stage of GV oocytes were not predictors of nuclear and cytoplasmic competence (10). We selected GV oocytes with prominent nuclei in homogenous cytoplasm with any type of defect in overall appearance of oocyte. The maturation and fertilization rates in our study show that these simple, practical criteria might be enough to choose GV for an extended culture program.

In this study, the means of percent of cleaving embryos that reached the four-cell stage in the IVM group approximated the *in vivo* group, but was not significantly different (p=0.529). While it was reported that day two embryos had reduced quality and four-cell embryos were significantly lower in the IVM group (28.3% vs. 54.3%, p=0.0026) (1), in a case report the cleavage rate was 71%, which resulted in a healthy baby (7). In another report, 84.5% of IVM cleaved embryos reached the four-cell stage after 40 hours and was not significantly different from the *in vivo* group, as seen with our study. The cleavage rate of the IVM group in our study and Kim's work did not significantly differ from the *in vivo* group in the four-cell stage (13). Additionally, Shu et al. reported after 4-6 hours of incubation, 100% of injected eggs that did not mature at the time of denudation were cleaved (14).

A comparison between IVM of oocytes in stimulated and unstimulated cycles reveals that without stimulation of gonadotrophins there is a reduction in collected oocytes and a subsequent decrease in cleavage and pregnancy rate. In fact, IVM is primarily used for women with PCO or PCOS to avoid OHSS. Regularly cycling women and over responders are other groups for HCG priming IVM (15). In PCOS patients there are significant variances in the reports of pregnancy and implantation rates among studies (17%-52% pregnancy; 6%-27% implantation) (15). In a recent study of 34 IVM cycles in PCO and PCOS cases, oocyte maturation and fertilization rates have been reported as 63% and 62%, respectively. The cleavage rate was 14.6% (all normal zygotes reached the two cell stage on day two) and only 9% day three embryos were of very good quality. The clinical gestation rate was 32%. Although the majority of the embryos (68%) were of a low quality, the pregnancy rate was comparable with classic stimulated cycles (16).

In another research on 63 regular cycles (568 oocytes with an average of 9) maturation rate, fertilization and cleavage rates were 74.3%, 72.6% and 89.0%, respectively (17).

The experience of somatic cell nuclear transfer to the ooplasm of GV oocytes (haploidization) resulted in subsequent extrusion of the first PB in 39.1% of reconstructed oocytes. This practice revealed that ooplasms in the GV stage probably play an essential role in the liberation of the first PB and nuclear maturation (18).

The results of this study and other works have shown variations in the fertilization and cleavage rates of IVM and *in vivo* MII oocytes. Additionally, the frequency of collected immature oocytes differs among studies. These dissimilarities might be due to the conditions under which studies have been performed. Several variables affect oocytes, therefore the variation between results is not unexpected. However, it seems the most important question pertains to which situations immature GV oocytes must be cultured. Can GV oocytes be ignored when there are few or no MII oocytes on the puncture day? Although cytoplasmic competency has been shown to be compromised in previous works, nuclear maturation in GV oocytes is a universal concept. According to our results and others, culturing GV oocytes should be performed in cases of few MII *in vivo* oocytes or in patients whose numbers of GV oocytes with respect to MII are high. There is no need to culture GV oocytes when there are enough mature oocytes on the puncture day.

## Conclusion

This study showed that prolonged culture of GV oocytes produced acceptable numbers of four-cell embryos on day two transfers. The developmental competence of GV-IVM oocytes were only defined in the early stage and did not significantly differ when compared with *in vivo* sibling oocytes.
